# Psychiatric aspects of burn

**DOI:** 10.4103/0970-0358.70731

**Published:** 2010-09

**Authors:** P. K. Dalal, Rahul Saha, Manu Agarwal

**Affiliations:** Department of Psychiatry, C.S.M. Medical University, Lucknow, Uttar Pradesh, India

**Keywords:** Anxiety, burn, depression, pain

## Abstract

Burn injuries and their subsequent treatment cause one of the most excruciating forms of pain imaginable. The psychological aspects of burn injury have been researched in different parts of the world, producing different outcomes. Studies have shown that greater levels of acute pain are associated with negative long-term psychological effects such as acute stress disorder, depression, suicidal ideation, and post-traumatic stress disorder for as long as 2 years after the initial burn injury. The concept of allostatic load is presented as a potential explanation for the relationship between acute pain and subsequent psychological outcomes. A biopsychosocial model is also presented as a means of obtaining better inpatient pain management and helping to mediate this relationship.

## INTRODUCTION

Psychological distress is among the most frequent and debilitating complications post-burn injury. Preliminary reports using the Burn Model System (BMS) dataset indicated that one-third of patients with major burns had clinically significant psychological distress at the time of discharge,[1] and the mean level of psychological distress in the BMS sample[2] was significantly higher than that reflected in published data from a normative sample.[3] In addition, psychological distress of in-patients of the hospital predicted significantly greater physical impairment for at least 1 year post-burn.[1] Clinically significant psychological distress also accounted for substantial variance in concurrently assessed quality of life at 2 (58%), 6 (68%), and 12 (51%) months post-burn injury.[4] Severe psychological distress is an important secondary complication of major burn injuries, with long-term consequences.

Stress disorders and depression are prevalent. For example, acute stress disorder (ASD) has been reported in 18–26% in Greek,[5] US,[6] and Dutch[7] samples. Post-traumatic stress disorder (PTSD) has been observed in one-third of Japanese[8] and US[6] samples between 3 and 6 months post-burn, and in 15–20% of Dutch[7] and Greek[5] samples at 1 year. PTSD was more common among veterans with extensive burns than among those with spinal injuries, amputations, major chest trauma, heart failure, or cardiac arrest.[9] In Australian sample, high levels of distress during a major brush fire was more strongly associated with PTSD symptoms than were sociodemographic or preexposure psychological variables.[10] Finally, clinically significant symptoms of depression were reported by 23% in a US sample[11] and 27% in a British sample[12] at 2 years; and 20% of Greek patients with burn injuries had a depressive disorder at 2 years.[5]

Distress may be manifested in other forms as well. Body image dissatisfaction appears common in patients with burn injuries.[13] Sleep disturbances occur frequently among in-patients with burns, e.g., nightmares in 39% and significant sleep problems in 75%.[14–16] Furthermore, many adult Swedish[17] and US[18] burn survivors continue to report nightmares (30–43%) and insomnia (37%) between 1 and 11 years post-burn. Sleep problems, PTSD symptoms, and scar-related problems were highly intercorrelated in a Dutch sample.[19]

Accumulating evidence suggests that psychological distress symptoms have a short- and long-term impact on health, function, and quality of life. Prolonged functional impairment has been associated with sleep disturbance,[20] subsyndromal PTSD,[18,21,22] depression,[11,23] body image dissatisfaction,[24] and syndromal PTSD.[6,25] The presence of such a wide range of symptoms and syndromes is not at all unexpected, given the plethora of stressors for patients with burns (e.g., burn event, losses, pain, repeated painful procedures, disfiguring injury, and unfamiliar surroundings).

## PSYCHIATRIC DIAGNOSES IN BURN PATIENTS

Until the release of Diagnostic and Statistical Manual of Mental Disorders, 4th ed., classificatory system (DSM-IV), the immediate aftermath of a traumatic event was accepted as a normal reaction to an overwhelming event. The conceptualisation of acute reactions to trauma as a normal response was supported by a widely held theoretical view of post-trauma reactions which suggested that alternating intrusive and avoidant PTSD symptoms represented normal information processing of an event overwhelming to one’s cognitive structures.[26]

### Acute stress disorder

The DSM-IV includes the diagnosis of ASD, which may be made as early as 3 days following the traumatic event.[27] Composed of dissociative, intrusive, avoidant, and arousal symptoms, the formulation of ASD emphasises dissociative symptoms. To be diagnosed with ASD, one must experience at least three of five possible dissociative symptoms but only one intrusive, avoidant, and arousal symptom. ASD was added to the DSM-IV, at least in part, on the basis of retrospective studies that documented the presence of dissociative symptoms including derealisation, depersonalisation, emotional numbing, and a reduction of awareness in one’s surroundings following various types of accidents.[28] Speigel and colleagues[28] have argued that a dissociative syndrome characterised by depersonalisation, derealisation, and psychic numbing is prominent immediately following a traumatic stressor.[28,29]

### Post-traumatic stress disorder

A diagnosis of PTSD requires the presence of at least one intrusive symptom and three avoidant and two arousal symptoms, each of which must persist for at least 1 month.[27] Three of the dissociative symptoms included in the ASD diagnosis (depersonalisation, derealisation, and time distortion/daze) are new to the DSM-IV; the other two (numbing, amnesia) have been previously classified as avoidant symptoms within the PTSD diagnosis. Burn injury has occupied a unique role in the trauma literature. Beginning with the work of Cobb and Lindemann in 1943[30,31] documenting acute psychological responses to the Cocoanut Grove fire, studies of burn injury have offered perspectives which have helped validate the idea that trauma has mental health consequences. In a study, Cobb and Lindemann described dissociation, re-experiencing, avoidance, and acute grief in those people hospitalised for burns following the Cocoanut Grove fire.[35] More recent studies have documented that up to 45% of adults who were hospitalised for their burn injury have PTSD 1 year later[32,33] and that severity of intrusive and avoidant PTSD symptoms within 1 week of injury predicts chronic PTSD.[34]

## PAIN, DEPRESSION, AND PHYSICAL FUNCTIONING FOLLOWING BURN INJURY

### Depression following burn

Much greater variability is found when outcomes beyond survivability are considered. For example, depression is well recognised as a significant problem following burn injury.[35] For most burn survivors, average scores on depression indices fall within the mild to moderate range.[36–38] However, moderate to severe symptoms of depression have been found in 18–45% of burn survivors, years after their physical injuries have healed.[38–40]

### Pain following burn

Pain is another serious problem for burn survivors, particularly during the early phases of burn care when open wounds are being subjected to debridement and movement therapies.[41] In addition, pain remains a concern for years after burn injury wounds have closed. Choniere and colleagues[36] found ongoing pain concerns in 35% of a sample of burn survivors, at least 1 year after injury. Similarly, Dauber *et al*.[42] found that 52% of burn survivors who were on an average of 10 years after injury reported the presence of pain. Of those with pain, 45% reported that pain interfered with their daily lives.[43] Malenfant and colleagues[43] found pain in over 36% of their sample and demonstrated that pain prevalence did not vary greatly between 1 and 4 years after injury. Although noting that the average severity of pain was mild (3.4 on a 0–10 visual analogue scale) among burn survivors, Malenfant and colleagues[49] point out that pain severity varied widely both within and between patients. For example, 19% of their sample reported average pain as severe.

### Association of pain, depression and functioning

Pain and depression represent suffering for burn patients, thus deserving attention in research and clinical settings. In addition, both pain and depression have been associated with other negative outcomes among burn patients. For example, two studies have demonstrated that elevated pain during hospitalisation for burn injuries is associated with poorer adjustment and reduced physical functioning up to 2 years after discharge from the hospital.[44,45] Depression has been associated with reduced physical function[40] and change in physical health over time[40] among burn patients. Although past studies have clearly shown that pain and depression have prospective associations with physical functioning, much less is known about how these conditions might interact as predictors of functioning among survivors of burn injuries. Cognitive–behavioural theories of pain, depression, and functioning have emphasised that certain cognitive processes associated with pain and depression may make the co-occurrence of these conditions especially deleterious to functioning. For example, persons with pain and depression show enhanced memory for negative self-referent pain and illness information as compared with persons with pain who are not depressed.[46] Vlaeyen and Morley[47] have noted that co-occurring pain and depression may activate cognitive processes that guide a person towards completing or terminating a task. For example, a person may terminate a functional activity as soon as he or she no longer enjoys the task, perhaps due to pain perceptions. Understanding associations between pain, depression, and physical functioning is critical because burn survivors have considerable difficulties in returning to personal, social, and community roles after their injuries have healed.[48,49]

## PSYCHIATRIC SEQUELAE OF BURN PAIN

In 1995, Ptacek *et al*.[45] focussed on procedural pain during hospitalisation as it is related to adjustment at 1 month post-discharge in 43 patients treated at a major regional burn centre. Using the Brief Symptom Inventory (BSI) and the Sickness Impact Profile (SIP), patients with higher pain scores (based on a composite of several measures over time) showed poorer adjustment on these measures after 1 month. The same research group looked at this question to see if the relationship held true longer than 1 month post-discharge and controlled for burn-related factors.[44] With a sample size of 122 burn survivors, they used the same pain composite scores (visual analogue scale pain ratings for in-patient procedural pain) to determine impact on adjustment at 1 and 2 years post-burn injury. The BSI, SIP, and a PTSD measure were again used to assess adjustment. Those with higher in-patient pain scores reported more symptoms on the BSI and SIP and higher rates of PTSD. At 2 years post-burn injury, higher in-patient pain scores correlated with PTSD symptoms.

Van Loey *et al*.[50] also found that anxiety related to acute pain was a predictor of PTSD symptoms, 1 year after the injury. In a later review of the literature, Van Loey and Van Son[51] concluded that the evidence was strong enough to suggest that patients who expressed high anxiety related to pain were at risk for developing PTSD symptoms long after discharge and stated that “prolonged, painful treatment of burn injuries enhances the risk of chronic PTSD.” An attempt was made by Saxe *et al*.[52] to establish a link between acute pain and long-term outcome after trauma by looking at the amount of morphine that was administered to children during their hospitalisation for burn injuries. They found that the amount of morphine received was indeed the best predictor of the future development of PTSD. Other studies have supported the relationship between chronic psychological and environmental stress and health and well-being in children in non-medical populations.[53] Edwards *et al*.[54] examined the relationship between acute pain at discharge and long-term suicidal ideation (SI). These investigators used the Bodily Pain Scale of the Short Form-36 and two items from the BSI that specifically assessed SI at 6 and 12 months post-burn injury. They also found that a patient’s prior mental health status and the characteristics of the injury, such as size of the burn, were poor predictors of later SI, which leads to the conclusion that pain severity was the most robust predictor for later SI. These findings are supported by previous evidence in the non-burn literature showing that there are disproportionately high suicide rates among individuals suffering from different types of pain. Specifically, studies have shown increased rates of SI in those who report persistent chronic pain conditions.[55–57]

### Allostatic load and the biopsychosocial model of adjustment

The concept of allostatic load can be useful in explaining the mechanisms of how high levels of acute pain can have an impact on a person, months to years later.

#### What is allostasis?

Sterling and Eyer[58] first defined allostasis as the adaptation that the body makes in response to stressful events. The process involves activation of several physiological systems, including the immune system, and is essentially the body’s ability to maintain “stability through change.” The body is able to cope effectively with these stressors when adaptations are activated infrequently; however, there is the potential for the system to become overloaded. McEwen and Stellar[59] described what happens to the body when these allostatic systems are overstimulated and were the first to use the term “allostatic load.” There are three types of allostatic load, including

1. frequent activation of allostasis,
2. the body’s inability to turn off allostasis when the stressor is removed, and
3. an inadequate response to the stressor.

McEwen[60] provides a more detailed discussion of allostatic load. Simply, it is the measure of cumulative wear and tear on the body. It is important to view allostatic load as an interaction between genetic, environmental, and social factors.

#### Studies validating the above theory

The identification of the body’s physiological response to stressors sparked a large surge of research examining the potential harmful effects of allostatic load on the body over time. Most relevant to the study of burn injuries is the body of literature that has shown slowed wound healing under psychological stress. Kiecolt-Glaser *et al*.[61] studied the amount of time it took a punch biopsy wound to heal in women who were in a typically stressful role of serving as caregivers for a demented relative and compared this with a control group of women in a less stressful environment, and found the wounds in the caregiver group took significantly longer to heal. This work was later replicated in a group of college students undergoing exams.[62] This relationship between high pain levels and delayed wound healing was maintained even after controlling for presurgery depressive symptoms and other post-surgical medical complications. It is hypothesised that the pain and wound healing link can be explained by the neuroendocrine and immune pathways that are altered under stress, and relevant to the process of wound healing. Specifically, the interactive effects of the glucocorticoids and proinflammatory cytokines are the primary physiological mechanisms underlying both stress and wound healing.[62] Although there have been no specific studies looking at the relationship between stress and the healing of burn wounds, these mechanisms are critical in restoring tissue perfusion, wound healing, and defending against infection, all of which are necessary in recovery from a burn injury. Patients with burn injuries are already susceptible to many types of infections due to the large areas of open wounds. These infections lead to failing skin grafts and longer lengths of hospitalisation. In patients who have large burns, infections can be life-threatening. If immune function is suppressed due to the stress of uncontrolled pain and depression, their ability to fight off infection is further compromised.

### Impact of biopsychosocial model in predicting outcome

As mentioned earlier, a person’s response to allostasis is a function of his or her personality style and coping mechanisms and how these interact over time with the environmental factors that are present. For example, the size and severity of a burn injury do not predict psychological outcome; factors such as whether the patient has a history of depression or alcohol abuse interact with coping style, social support, secondary complications, and pain, to determine outcome. This is otherwise known as a biopsychosocial model, and it can be very useful in explaining and predicting the long-term outcome of burn injuries. Acknowledging that outcome depends on such a complex interplay of factors can enable us to understand why a person with minor burns may show a devastating psychological reaction, whereas someone with a massive burn injury may adjust surprisingly well.

## HANDLING PSYCHOSOCIAL ASPECTS OF BURN INJURIES

With the increased survival of patients with large burns comes a new focus on the psychological challenges and recovery that such patients must face. Most burn centres employ social workers, vocational counsellors, and psychologists as part of the multidisciplinary burn team. Physiological recovery of burn patients is seen as a continual process divided into three stages: resuscitative or critical, acute, and long-term rehabilitation.[63] The psychological needs of burn patients differ at each stage.

### Resuscitative or critical stage

The psychological characteristics of this stage include

- Stressors of the intensive care environment, uncertainty about outcome, and a struggle for survival.
- The intensive care environment can be both overstimulating and understimulating with the monotony of lying in a hospital bed for weeks.
- Cognitive changes such as extreme drowsiness, confusion, and disorientation are common during this phase.
- More severe cognitive changes such as delirium and brief psychotic reactions also occur, usually as a result of infections, alcohol withdrawal, metabolic complications, or high doses of drugs.
- Patients may also be intubated, which greatly limits direct communication.

#### Treatment

- Patients should be encouraged to cope with the frighteningly unusual circumstances of the intensive care unit through whatever defences are available to them, even primitive strategies such as denial and repression.
- Supportive psychological interventions should focus on immediate concerns such as sleep, pain control, and protecting patients’ coping strategies.
- Non-pharmacological approaches to pain control, such as hypnosis and relaxation, can be effective.
- Educate and provide support to family members.
- Educate and provide support to staff.

### Acute stage

The acute phase of recovery focusses on restorative care, but patients continue to undergo painful treatments. As patients become more alert during this phase, they face these procedures with less sedation. Also, patients are more aware of the physical and psychological impact of their injuries. The psychological characteristics of this stage include

- Depression,
- Anxiety,
- Sleep disturbance,
- Pain,
- Grief
- Premorbid psychopathology –
  - Patients with pre-existing psychopathology typically cope with hospitalisation through previously established dysfunctional and disruptive strategies.
  - The most common premorbid psychiatric diagnoses are depression, personality disorders, and substance misuse.
  - Prior psychopathology can have an adverse impact on outcomes, including longer hospitalisations and the development of more serious psychopathologies after injury.

#### Treatment

- Drug management of anxiety, sleeplessness, and depression
- Brief counselling
- Pharmacological management of pain
  - Long-acting opiates are used for background pain
  - Short-acting opiates are used for painful procedures such as wound care
- Teach non-drug approaches to pain management
  - Relaxation
  - Imagery
  - Hypnosis
  - Virtual reality
  - Cognitive behaviour therapy

### Long-term rehabilitation

The long-term stage of recovery typically begins after discharge from hospital, when patients begin to reintegrate into society. For patients with severe burns, this stage may involve continued outpatient physical rehabilitation, possibly with continuation of procedures such as dressing changes and surgery. This is a period when patients slowly regain a sense of competence while simultaneously adjusting to the practical limitations of their injury. The psychological characteristics of this stage include the following.

- *Physical*—Itching, limited endurance, decrease in functioning
- *Social*—Changing roles, return to work, body image, sexual issues
- *Psychological*—Anxiety, depression

#### Treatment

- Hospital-based programme for image enhancement
- Outpatient counselling
- Social skills training
- Support groups
- Peer counselling
- Vocational counselling

## CONCLUSIONS

A burn injury and its subsequent treatment are among the most painful experiences a person can encounter. The emotional problems experienced by people suffering burn injuries, who number in the hundreds of thousands, have been largely ignored. The emotional needs of patients with burns have long been overshadowed by the emphasis on survival. Patients undergo various stages of adjustment and face emotional challenges that parallel the stage of physical recovery. Adjustment to a burn injury seems to involve a complex interplay between the patient’s characteristics before the injury, moderating environmental factors, and the nature of the injury and ensuing medical care. Understanding the process behind the mental suffering in a patient following burn will help the clinicians to recognise the psychiatric sequelae. This will help in providing appropriate psychiatric services to the patients of burn and will help in speeding up their recovery. Proper rehabilitation into their social, occupational and family situation will thus be more easily achievable and emotional needs of the patient can be handled more effectively.
